# Zoonotic Anthrax Outbreak in Bangladesh: An Urgent Call for an Integrated One Health Control Strategy

**DOI:** 10.1002/hsr2.72240

**Published:** 2026-03-29

**Authors:** Hemayet Hossain, Md. Shahidur Rahman Chowdhury, Md. Mahfujur Rahman

**Affiliations:** ^1^ Department of Microbiology, Faculty of Health Sciences Gono Bishwabidyalay Ashulia Bangladesh; ^2^ Department of Veterinary Science and Animal Husbandry Teesta University Rangpur Bangladesh; ^3^ Department of Medicine Sylhet Agricultural University Sylhet Bangladesh

**Keywords:** anthrax outbreaks, cutaneous anthrax, one health, livestock vaccination, zoonotic disease control

## Abstract

**Background:**

Bangladesh continues to experience recurrent zoonotic anthrax outbreaks, particularly in livestock‐dense districts where informal slaughtering and gaps in carcass disposal persist. In August–October 2025, a multi‐upazila (sub‐district) outbreak was reported in Rangpur and Gaibandha district, raising renewed public health concern.

**Methods:**

This perspective summarizes preliminary surveillance data published by the Institute of Epidemiology, Disease Control and Research (IEDCR), field investigations of local veterinary hospitals, and clinical observations. Human cases were diagnosed based on characteristic cutaneous lesions, epidemiological links to infected animals, and confirmatory bacterial culture of *Bacillus anthracis* from selected samples.

**Results:**

More than 200 human cutaneous anthrax cases, including two fatalities (in Rangpur), were documented alongside over 200 livestock deaths in Rangpur and Gaibandha, Bangladesh. Most outbreaks were associated with the slaughtering and consumption of meat from sick animals, while improper carcass disposal likely contributed to environmental contamination. The outbreak disrupted rural markets, threatened animal health, and underscored persistent diagnostic limitations at the community/upazila level veterinary hospitals.

**Conclusion:**

The 2025 anthrax event highlights entrenched vulnerabilities within animal health infrastructure, slaughter regulation, and public awareness. Strengthening mass vaccination, coordinated surveillance, safe carcass disposal, and risk communication through an integrated One Health approach is essential to interrupt transmission cycles and prevent environmental persistence of *B. anthracis* spores in Bangladesh.

## 1 Introduction

The recurrent anthrax (Known as “Torka” in Bangladesh) outbreaks in Bangladesh pose serious public health concerns. Anthrax, caused by *Bacillus anthracis* (a spore forming gram positive bacterium), is a resilient zoonosis capable of persisting for decades through durable spores, making it a constant threat at the human ‐ animal ‐ environment interface [1]. Human anthrax can occur in four clinically distinct forms, each differing in severity and incubation based on the route of entry [2]. These include cutaneous anthrax, typically appearing within 1–12 days characterizing painless necrotic skin lesion with a characteristic black eschar and surrounding edema [3]; inhalational anthrax, which may take 1–60 days to manifest is characterized hemorrhagic mediastinitis with mediastinal lymph node necrosis and pleural effusion [4]; gastrointestinal anthrax characterizing ulcerative hemorrhagic lesions of the intestinal mucosa with mesenteric lymph node necrosis, developing over 1–6 days following ingestion of contaminated meat; and injectional anthrax, emerging 1–10 days after exposure are characterized extensive soft‐tissue necrosis, edema, and hemorrhage at the injection site without eschar formation [5]. In animals, peracute and acute anthrax are characterized by widespread hemorrhages, marked splenomegaly with a “blackberry jam” appearance, and dark, unclotted blood oozing from natural orifices, whereas subacute and chronic forms present with localized edema, hemorrhagic lymphadenitis, and necrotic lesions of the gastrointestinal tract [4]. Of these, the cutaneous presentation is by far the most common, representing more than 95% of reported cases and generally arising from direct contact with infected animals or contaminated animal products [6].

Historically, the largest anthrax outbreak in Bangladesh occurred during 2009–2010, when a national red alert followed more than 600 human cutaneous cases across multiple districts, driven by informal slaughtering, poor carcass disposal, and low vaccination coverage highlighting a persistent failure to move beyond reactive outbreak control [1]. Bangladesh, with dense livestock populations, informal meat markets, and limited veterinary resources, has become a hotspot for outbreaks. The disease typically emerges during periods of rainy seasons or heavy rainfall or flooding, which can expose buried spores of *B. anthracis* and contaminate pasture lands [7]. Inappropriate practice such as slaughtering moribund animals for consumption further amplify transmission risk in rural communities [8, 9]. Usually, people unaware about the moribund nature of slaughtered animals in Bangladesh [10]. Although slaughter regulations in Bangladesh strictly prohibit the slaughter of moribund animals, animals injured without veterinary authorization, animals affected by infectious diseases, and pregnant animals, these regulations are frequently violated in practice [11]. When cattle become critically ill and fail to respond to treatment, farmers often sell them at a low price to slaughterhouse workers to minimize economic loss. Consequently, such sick or moribund animals are slaughtered and enter the food chain [9, 12]. This practice represents a common field scenario in Bangladesh and has been implicated in nearly all reported anthrax outbreaks, which are largely associated with the slaughtering of sick and moribund animals.

Additionally, inadequate carcass disposal, slaughtering sick animals, weak monitoring of slaughter house activity and weak diagnostic surveillance at the field level contribute to ongoing outbreaks. Moreover, limited public awareness regarding recognition of cutaneous lesions, delayed treatment seeking, and improper handling of meat perpetuate preventable morbidity [13]. Environmental persistence of spores in endemic districts creates a cycle conducive to recurring outbreaks, highlighting the urgent necessity for comprehensive and sustained interventions. This perspective aimed to (i) summarize the ongoing anthrax situation in Bangladesh using recent surveillance and diagnostic evidence, (ii) identify key drivers sustaining recurrent outbreaks, and (iii) propose an integrated One Health control strategy focused on surveillance, prevention and rapid response.

## 2 Recent Outbreak of Zoonotic Anthrax

**Figure 1:**
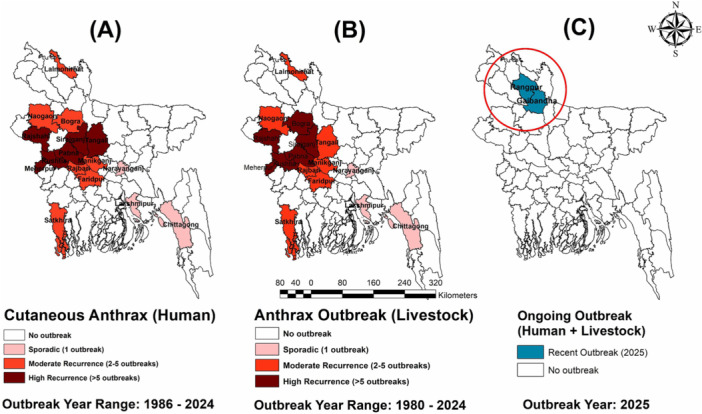
Geographic distribution of anthrax outbreaks both in human and animal in Bangladesh from 1980 to 2025. (A) Human cutaneous anthrax outbreaks reported between 1986 and 2024. (B) Livestock anthrax outbreaks reported between 1980 and 2024. (C) The 2025 ongoing outbreak in Rangpur and Gaibandha district is marked in Circle. This choropleth map is generated using ArcMap 10.8 software.

The most recent outbreak in Pirgacha, Kaunia and Mithapukur upazila of Rangpur and Sundarganj upazila of Gaibanda (August–October 2025) once again underscores the vulnerability of the country (Figure 1). Reports indicated more than 200 cutaneous anthrax cases in humans, with two fatalities, alongside the deaths of over 200 cattle and goats [14]. Notably, the IEDCR also investigated the presence of *B. anthracis* in frozen beef from Rangpur district [15]. Alarmingly, carcasses of infected animals were slaughtered, and meat was sold for consumption, creating a cycle of continued exposure [10]. After infected carcasses are exposed to air, *B. anthracis* bacilli are released and later form spores in the environment, thereby increasing the risk of persistent contamination.

### 2.1 History, Epidemiology and Transmission Dynamics

The epidemiology of anthrax in Bangladesh demonstrates a recurring pattern of outbreaks affecting both livestock and humans. Beyond direct animal contact, anthrax persistence in Bangladesh is reinforced by long‐term survival of *B. anthracis* spores in alkaline soils, with monsoon‐driven flooding and soil disturbance facilitating spore resurfacing and re‐exposure [16]. Incorporating GIS‐based hotspot mapping and flood‐risk modeling could strengthen outbreak prediction and targeted prevention in endemic districts.

Sporadic anthrax outbreaks were reported in the 1980s, affecting livestock and resulting in several animal deaths [1]. The disease re‐emerged dramatically in 2009–2010, prompting the Institute of Epidemiology, Disease Control and Research (IEDCR) to issue a national red alert [1, 5] (Table 1). More than 600 human cases were recorded, predominantly in cattle‐rich districts such as Pabna, Sirajganj, Kushtia, and Meherpur, alongside numerous livestock deaths that severely impacted rural livelihoods [1].

**Table 1:** Historical anthrax outbreaks in Bangladesh (1980–2025).

Year (period)	Location (districts/area)	Affected host	No. affected (Humans/Animals)	No. dead (Humans/Animals)	References
1980–1984, 1986	Multiple districts (historical reports)	Humans and livestock	Reported outbreaks (*n* = 1) only. Livestock: 62, Humans: 27 (Cutaneous form)	Livestock: 43	[1]
2008–2009 (DLS registry)	Nationwide reporting (higher in cattle‐rich districts)	Livestock	DLS registered 437 (2008) and 449 (2009) animal anthrax cases.	Not specified	[5]
2009–2010 (re‐emergence/explosive 2010 outbreaks)	Pubna, Sirajganj, Kushtia, Meherpur, Tangail, Manikganj, Satkhira, Lalmonirhat, Rajshahi, Narayanganj, Laxipur, and Chattogram	Humans (cutaneous) and livestock	Humans: reported 607 cases (2010 national red alert). Livestock: field investigations reported dozens to hundreds (e.g., 104 animal cases over July–Sept 2010 in one review).	Not specified	[1, 10]
2011	Sirajganj, Pabna, Bogra, Meherpur, Tangail, Faridpur (multi‐district clusters)	Humans (cutaneous) and livestock	Humans: 122 suspected human cases (2011 outbreaks). Animals: 1668 livestock cases reported to DLS in association with these outbreaks.	Humans: 2 human deaths reported (two suspected anthrax fatalities). Animals: 173 animal deaths recorded.	[17, 18]
2012	Sirajganj, Tangail, Pabna	Humans and livestock	Humans: 449, Livestock: 140 in 14 outbreaks	Livestock: 98	[17, 19]
2013–2016 (clustered outbreaks)	Seven districts (Meherpur, Sirajganj, Kushtia, Naogaon, Rajshahi, Bogura, Pabna)	Humans (cutaneous anthrax) and livestock	Humans: a total of 1210 suspected cutaneous anthrax cases identified across 26 outbreaks (2013–2016). Animals: in 16 investigated outbreaks, 5937 affected livestock	Humans: Not specified. Animals: variable; 801 livestock died	[20, 21]
2016–2017 (selected outbreaks and investigations)	Sirajganj, Tangail, Rajbari (Kalukhali) and other risky districts	Humans and livestock	Humans: pooled reports indicate 70 people infected across three outbreak sites (2016–17 investigations). Specific: Kalukhali (Rajbari) outbreak investigation documented 17 people with skin lesions following slaughter of an ill animal. Animals: a number of animal anthrax cases were confirmed at the same sites.	Humans: no large fatalities reported in these specific small site investigations (cases were treated). Animals: several livestock deaths documented at outbreak sites (case counts reported in field reports).	[17]
2017 (Kalukhali, Rajbari) specific investigation	Kalukhali upazila of Rajbari district	Humans and livestock	Humans: 17 people with cutaneous lesions from one local outbreak linked to slaughtering an ill cow. Animals: the implicated cross‐breed cow and local livestock deaths described.	Humans: treated; deaths not reported in that investigation. Animals: local animal deaths associated with the event.	[20, 22]
2010–2017 (cumulative view)	Multiple (16 districts overall)	Humans and livestock	Between 2010 and 2017, repeated outbreaks resulted in 2581 suspected human cutaneous anthrax cases reported to national surveillance. Animal case totals across those years amount to several thousand reported in DLS registries (varies by year).	Human deaths: relatively uncommon in reported cutaneous outbreak investigations; animal fatalities were substantial in some years (hundreds in some annual reports).	[20]
2022	Sirajganj and Meherpur	Human (cutaneous)	Out of 464 suspected cases, 85 samples were tested of which 62 were positive (73%)	None	[13]
2023	Meherpur	Human (cutaneous)	Out of 655 suspected cases, 142 samples could be tested of which 78 (57%) were positive	None	[13]
2025 (August – September)	Pirgacha, Kaunia and Mithapukur upazila of Rangpur and Sundarganj upazila of Gaibanda	Humans (cutaneous) and livestock	Humans: local media reports state > 200 people with anthrax‐like skin lesions (affected after handling sick animals or meat). Livestock: Widespread animal morbidity	Human: 2 human deaths Animals: media report > 200 cattle and goats died in the area (locals/livestock office quoted).	[10, 14]

In 2011, multi‐district outbreaks led to 122 suspected human cases, nearly 1700 livestock cases, and a combined total of 175 human and animal deaths [17, 18]. From 2013 to 2016, at least 1210 human infections were identified across 26 outbreaks, while close to 6,000 animals were affected, with over 800 deaths [22]. A localized outbreak in 2017 in Kalukhali, Rajbari, infected 17 individuals after slaughtering sick cattle [17]. Since 2009, approximately 2400 suspected human anthrax cases have been reported from 36 outbreaks across 16 districts of Bangladesh, with the majority occurring in Sirajganj and its neighboring districts. Most anthrax outbreaks in Bangladesh typically occur between May and November [23].

These repeated outbreaks underline endemicity, systemic weaknesses in vaccination, slaughter regulation, and carcass disposal, with the 2025 outbreak in Rangpur and Gaibandha districts representing a continuation of this entrenched problem.

### 2.2 Diagnosis of Zoonotic Anthrax

The reported human cases were first suspected based on the presence of a painless papule progressing to a vesicle and ulcer with a characteristic black eschar, surrounded by marked non‐pitting edema. Diagnosis of human anthrax (Cutaneous) by the Institute of Epidemiology, Disease Control and Research (IEDCR), was based on clinical evaluation of typical eschar‐forming skin lesions with epidemiological linkage to infected livestock, supported initially by smear staining and bacterial culture, and subsequently confirmed by RT‐PCR testing. In animals, local veterinary hospitals of Rangpur and Gaibandha assessed sudden death without rigor mortis, dark unclotted blood, and hemorrhagic discharges. Field history, carcass assessment, and exclusion of differential diagnoses strengthen case confirmation.

### 2.3 Contributing Factors and Epidemiological Associations to Anthrax Outbreak

The recurrent anthrax outbreaks reflect the fragmented control system in Bangladesh. The restricted and reactive nature of vaccination leaves livestock at risk. Sick animals are often slaughtered without regulation, and the meat is sold in local markets. Unsafe carcass disposal methods contaminate soil and water, as remains are often discarded in the fields or rivers. Cutaneous lesions are frequently misidentified due to poor public awareness. Confirmation takes long time due to inadequate diagnostic facilities in rural level. These systemic gaps enable outbreaks to recur, cases to go unreported, and re‐emergence.

The persistent recurrence of anthrax in Bangladesh point to the necessity for a comprehensive One Health‐driven control measures integrating public health, veterinary, and environmental systems.

**Figure 2:**
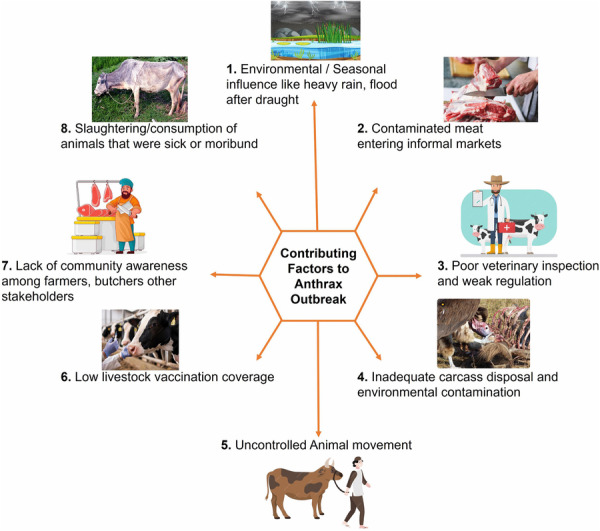
Probable factors contributing to the recent outbreak of Zoonotic anthrax in Rangpur and Gaibandha districts.

Through the outbreak hotspot investigations, we identified the total of 08 (Eight) probable causes of recent outbreak in 2025 (Figure 2).

### 2.4 Economic Impact of Anthrax

The economic burden is severe, as livestock deaths reduce meat, milk, and draft power, while fear of contaminated meat disrupts markets [24]. During the 2010 anthrax panic, the livestock sector in Bangladesh suffered an estimated financial loss of BDT 3200 crore (USD 370 million) due to reduced cattle sales, disrupted meat markets, and losses borne by farmers, traders, and butchers.

Reports confirm a significant anthrax outbreak in Rangpur and Gaibandha in 2025, with multiple human cases and widespread livestock infections, indicating likely economic impacts through animal loss and market disruption [10].

### 2.5 Integrated One Health Control Model

We propose a comprehensive four‐pillar One Health framework for zoonotic anthrax control in Bangladesh. The first pillar, Surveillance and Early Detection, emphasizes strengthening veterinary and human reporting systems, training frontline workers, and expanding diagnostic capacity. The second, Immediate Response, focuses on enforcing quarantine, banning slaughter of suspected animals, ensuring safe carcass disposal, and providing prompt treatment to exposed individuals. The third, Prevention, prioritizes mass livestock vaccination, distribution of protective equipment, and awareness campaigns for at‐risk communities. The fourth, Long‐Term Sustainability, highlights mapping endemic areas, integrating anthrax into national health programs, advancing research on spore ecology, and building veterinary and community resilience.

The One Health control model also incorporate participatory epidemiology by engaging farmers, butchers, and community stakeholders in early disease recognition, outbreak reporting, and hotspot identification. This community‐based approach strengthens surveillance, improves risk communication, and supports sustainable anthrax control.

The integrated control model (Table 2) aligns these pillars with measurable indicators, ensuring coordinated actions across animal, human, and environmental health systems for lasting success. Collaboration with WHO, FAO, and OIE remains crucial for lasting success.

**Table 2:** One health integrated control model for zoonotic anthrax, demonstrating sector‐specific interventions, intended outcomes, and monitoring indicators.

Sector	Key interventions	Intended outcome	Monitoring
Animal health	1. Mass and ring vaccination of livestock using adaptive delivery models (integration with agricultural extension services, mobile veterinary units in remote areas) 2. Active case finding in herds 3. Quarantine and restriction of animal movement in outbreak zones 4. Safe slaughtering practices 5. Cold‐chain strengthening through solar‐powered cold boxes and district vaccine hubs 6. Veterinary treatment or supportive care	1. Prevent recurrence and spread in animals 2. Break transmission chain at the source 3. Reduce livestock mortality and economic loss	1. % livestock vaccinated in outbreak zone 2. Number of suspected animal cases detected 3. Compliance with movement restrictions
Environment	1. Safe carcass disposal (burning/incineration) 2. Deep burial (≥ 2 m) with lime treatment in fuel‐limited rural settings 3. Avoid disturbing old burial sites 4. Soil hotspot mapping and surveillance 5. Restrict grazing on contaminated pastures 6. Vector/scavenger control 7. Exploration of biological soil decontamination approaches (e.g., bacteriophage‐based methods) in pilot settings	1. Minimize environmental contamination 2. Prevent spore persistence and re‐exposure 3. Reduce long‐term ecological risk	1. Number of carcasses safely disposed 2. Number of hotspot sites mapped and restricted 3. Evidence of spore reduction in soil tests
Human health	1. Early case detection and wound care 2. Immediate antibiotic PEP 3. Risk communication and awareness 4. PPE use for butchers, farmers, handlers 5. Clinical management and referral	1. Reduce human morbidity and mortality 2. Prevent secondary cases 3. Build trust in health system response	1. Number of human cases reported weekly 2. % exposed persons receiving PEP 3. PPE availability and usage rate
One health coordination	1. Joint animal–human surveillance 2. Strengthening lab diagnostic capacity 3. Rapid response teams (RRTs) 4. Risk communication strategies 5. Vaccine, antibiotic, PPE supply chain 6. Integration of outbreak data with GIS‐based hotspot mapping and flood‐risk assessment	1. Integrated outbreak response 2. Data‐driven decisions 3. Efficient resource use 4. Sustainable anthrax control	1. Time from case report response (hours) 2. Number of joint investigations conducted 3. Availability of supplies in field stock

## 3 Conclusion

The 2025 anthrax outbreak in Rangpur and Gaibandha resulted in over 200 human cutaneous cases, two fatalities, and more than 200 livestock deaths, predominantly linked to the slaughter of sick animals and improper carcass disposal. These findings demonstrate persistent gaps in vaccination coverage in Rangpur and Gaibandha districts, regulatory enforcement, and community awareness. Evidence from outbreak investigations indicates that unsafe slaughtering practices and environmental contamination are key drivers of recurrent anthrax in Bangladesh. Implementing a coordinated One Health approach integrating mass livestock vaccination, safe carcass management, early human case detection, and risk communication is essential to interrupt transmission, reduce human and animal morbidity, and mitigate economic losses in endemic regions. This outbreak underscores that anthrax remains a preventable yet recurring threat, necessitating sustained, context‐specific interventions.

## Author Contributions

**Hemayet Hossain:** conceptualization, methodology, software, data curation, writing – original draft, writing – review and editing. **Md. Shahidur Rahman Chowdhury:** methodology, data curation, investigation, writing – original draft. **Md. Mahfujur Rahman:** conceptualization, methodology, software, data curation, writing – original draft, writing – review and editing, supervision, resources. All authors have read and approved the final version of the manuscript. **Md. Mahfujur Rahman** had full access to all of the data in this study and takes complete responsibility for the integrity of the data and the accuracy of the data analysis.

## Funding

The authors have nothing to report.

## Conflicts of Interest

The authors declare no conflicts of interest.

## Transparency Statement

The lead author Md. Mahfujur Rahman affirms that this article is an honest, accurate, and transparent account of the study being reported; that no important aspects of the study have been omitted; and that any discrepancies from the study as planned (and, if relevant, registered) have been explained.

## Data Availability

The authors have nothing to report.
